# Subdose of human chorionic gonadotropin applied at the *Hou Hai* acupoint on follicular dynamics and luteal development in donkeys

**DOI:** 10.1590/1984-3143-AR2020-0554

**Published:** 2020-11-25

**Authors:** Márcio de Oliveira Ribeiro, Rodrigo Freitas Bittencourt, Marcus Antônio Rossi Feliciano, Ana Lúcia Almeida Santana, Mariana Alves de Andrade Silva, Morgana Duarte Felix, Larissa Rodrigues Santana, Larissa Pires Barbosa

**Affiliations:** 1 Universidade Federal do Recôncavo da Bahia, Centro de Ciências Agrárias, Ambientais e Biológicas, Cruz das Almas, BA, Brasil; 2 Universidade Federal da Bahia, Escola de Medicina Veterinária e Zootecnia, Salvador, BA, Brasil

**Keywords:** acupuncture, donkey, doppler, progesterone, pêga race

## Abstract

The objective of this study was to evaluate the effects of an hCG subdose applied at the *Hou Hai* acupoint as an ovulation inducer in donkeys. Eleven donkeys were distributed in randomized blocks in T1 = application of 1,500 IU of hCG intravenous (IV); T2 = 450 IU of hCG applied at the false acupoint (IV), and T3 = 450 IU of hCG applied at the *Hou Hai* acupoint. There was no difference (P > 0.05) between the treatments regarding the mean diameter of the pre-ovulatory follicle (34.5 ± 1.3 mm), the ovulation rate (96.97%), the interval between induction and ovulation (58.07 ± 16.82 h), the mean diameter of the CL (D0 = 23.0 ± 0.6; D2 = 27.7 ± 1.9 and D8 = 28.2 ± 0.8mm), and serum P_4_ concentrations (10.50 ± 2.99 ng.mL^-1^). The application of 450 IU of hCG at the *Hou Hai* acupoint increased ovulation rate (72.73%) more than 48 h after induction (P = 0.03) and a larger diameter of the CL on D4 (30.7 ± 5.1 mm) (P = 0.04). The vascularization area of the CL on D8, obtained by minimum number of colored pixel (NCP), was greater (P < 0.05) in the donkeys that received 1,500 IU of IV hCG (T1, 41.91 ± 1.17), and we found a positive correlation (P < 0.05) between mean NCP and P_4_ concentration in the donkeys that received 450 IU of hCG IV at the false acupoint (T2) or at the *Hou Hai* acupoint (T3). The application of 450 IU of hCG by IV route at the false acupoint or the *Hou Hai* acupoint was sufficient to induce ovulation in donkeys, demonstrating that the average dosage commonly used for this species is too high.

## Introduction

Because they are rustic animals and adapted to semi-arid regions, donkeys are often used in agricultural work, which is perhaps the most important role played by donkeys. However, few studies have been carried out to elucidate the reproductive physiology of this species, which is mistakenly compared to that of equines (Taberner et al., 2008; Tadesse and Tefera, 2013). As such, some researchers have focused specifically on the physiological aspects of the reproductive cycle of donkeys, especially after the development of conservation programs aimed at endangered races of donkeys and populations at risk of extinction (Mariante and Egito, 2002; Galisteo and Perez-Marin, 2010).

As with the equine species, human chorionic gonadotropin (hCG) is used in donkeys as an ovulation inducer, because it is similar to luteinizing hormone (LH) (Wilson et al., 1990) and because it can promote an ovulation rate as high as 100% up to 48 hours after application (Awan et al., 2016), as well as elevating progesterone (P_4_) production after ovulation (Kohne et al., 2014). However, hCG is commonly applied intravenously (IV), intramuscularly (IM), or subcutaneously (SC) (Romano et al., 2015), and the dose used in protocols for donkey species is the same as that indicated by the manufacturers for mares.

An alternative route for hormonal application is pharmacopuncture, which enables the use of reduced doses and has similar results to those obtained using conventional doses (Luna et al., 2008; Souza et al., 2019; Cardoso et al., 2018). Pharmacopunture also reduces undesirable side effects, sediments in animal products for consumption, and treatment costs (Wynn et al., 2001), thus enabling successive applications during the breeding season (Gastal et al., 2006). The *Hou Hai* acupoint, also known as Chang Qiang (Governing Vessel 1 - GV1), has a sedative indication (Luna et al., 2008), and is one of the points that is linked to the reproductive organs, as well as being easily accessible (Wang et al., 2007), making it viable to apply hCG to this acupoint in the field.

However, studies on the use of acupoints and reduced doses of hormones in ovulation induction protocols are still rare, especially in donkey species, as previously discussed. Thus, the goal of this study was to evaluate the effectiveness of subdoses of hCG administered at the *Hou Hai* acupoint as an ovulation inducer in donkeys.

## Material and methods

### Study location and ethical approval

The study was performed at the Experimental Farm of the School of Veterinary Medicine and Animal Science of the Federal University of Bahia (EMVZ/UFBA), in the city of Entre Rios-BA, located at a latitude of 11º56'31” south, a longitude of 38º05'04” west, and an altitude of 152 meters above sea level. The climate is tropical, with a dry season in the summer, according to the Köppen-Geiger climatic classification. The average annual rainfall is 1,550 mm, and the mean temperature is 24.0 °C, with a minimum of 16.8 °C and a maximum of 32.5 °C.

The project was approved by the Ethics Committee on Animal Use (CEUA) of EMVZ / UFBA, under registry nº 67/2016.

### Animals and experimental design

Eleven reproductively active female donkeys of the Pêga breed were used in this study. All donkeys were considered healthy after physical and obstetric examination, and their body condition score ranged between 4 and 6. The donkeys were used in three periods, distributed in randomized block crossover designs, with the period used as the blocking factor. There were three treatments, as follows: T1: 1,500 IU of hCG (Chorulon, MSD, Brazil, 100% dosage) applied intravenously (IV) in the jugular vein; T2: 450 IU of hCG (30% dosage) applied IV in the jugular vein; and T3: 450 IU of hCG (30% dosage) applied at the *Hou Hai* acupoint. There were a total of 11 replicates per treatment, and each donkey was considered an experimental unit.

### Ovulation inducer protocol

All donkeys were monitored every 48 h using mode-B ultrasonographic (US) examination. The first ovulation of the reproductive season was not included in the study to avoid the transition phase, and we confirmed the reestablishment of reproductive activity in the mating season. Eight days after the first ovulation, 5 mg of dinoprost tromethamine (Lutalyse, Pfizer, USA) was administrated to all donkeys by IM route, as a luteolytic agent, followed by daily monitoring of follicular development by B-mode US examination. When a dominant follicle presented with a diameter ≥ 30 mm and we observed the presence of grade 3 uterine edema, ultrasound examination of the pre-ovulatory follicle was performed in color Doppler mode to evaluate perifollicular vascularization. Ovulation was induced with hCG, using the dose and route of each specific treatment.

Previous antisepsis was performed on IV hCG applications in the jugular and *Hou Hai* acupoint with 70% alcohol. For applications in the *Hou Hai* acupoint, located in the depression between the average distance from the base of the tail (coccygeal muscle) to the anal sphincter with innervation in the caudal rectal nerve, a 1mL syringe was used, coupled to a 16G catheter, inserted in an angular position of 45°, with a 45mm depth (catheter's size) (Hwang and Limehouse, 2006).

### Evaluated variables

The variables evaluated in this study were: mean diameter (mean of the larger horizontal measure and the larger vertical measure) and vascularization (number of colored of minimum, maximum, and mean, and heterogeneity) of the pre-ovulatory follicle and CL on days 0 (D0), 2 (D2), 4 (D4), and 8 (D8); ovulation rate in the first 48 h after induction; interval between induction and ovulation; ovulation rate at 48 h after induction; serum concentration of P_4_ on D8; and the correlation between mean CL diameter, P_4_ concentration, and blood flow of the pre-ovulatory and CL follicles.

After the ovulation inducer was applied, B-mode ultrasonography evaluations were performed twice a day, at 12-h intervals, until the approximate time of ovulation, which is associated with the disappearance of the follicle image (anechoic) and replacement by the image of the CL (hypoechogenic). To follow CL development, measurements of the mean diameter in B-mode and Doppler vascularization were performed on D0 (day of ovulation); D2; D4, and D8. A color Doppler ultrasonography device (Mindray Z5, model DP 2200 VET, China) coupled to a linear transrectal transducer (5.0 Hz) was used to assess blood perfusion (follicular and luteal vascularization), visualizing color changes corresponding to blood flow throughout the follicular wall and luteal parenchyma.

The pre-ovulatory follicle and CL images obtained by color Doppler US were analyzed using Image ProPlus software (Media Cybernetics Inc., San Diego, California, USA) at the Laboratory of Ultrasonography of the Animal Reproduction Sector of the Faculty of Agrarian and Veterinary Sciences of the Paulista State University “Júlio de Mesquita Filho”. A tool within the software was used to circumvent the vascularized areas of the pre-ovulatory follicle and CL in order to obtain the average number of colored pixel [NCPs; ecotextures data, pixel heterogeneity (standard deviation NCPs)], as well as minimum and maximum NCPs.

Serum P_4_ concentrations were obtained from blood samples collected by jugular vein puncture, using tubes for vacuum collection without anticoagulant, eight days after ovulation. After collection, the blood was centrifuged (300 x g for 15 minutes) to obtain the serum, which was transferred to polypropylene microtubes with a capacity of 2 mL. Samples were labelled, stored at -20 ºC, and sent for analysis to the Animal Reproduction Laboratory of the Department of Animal Science of the Federal University of Viçosa. The chemiluminescence method was used with a commercial kit (Access Progesterone, Beckman Coulter, California, USA) for P_4_ concentrations, following the manufacturer’s guidelines.

### Statistical analyses

The data were evaluated for normality using the Shapiro-Wilk test, followed by variance analysis, and Tukey test for the parametric data (mean diameter and follicle vascularization at the moment of induction, interval between inducer application and ovulation, mean diameter, and CL vascularization on D0, D2, D4, and D8). We used the Kruskal-Wallis test for non-parametric data (ovulation rate before and after 48 hours of inducer application). To evaluate correlations between mean CL diameter, serum P_4_ concentrations, and pre-ovulatory follicle and CL blood flow on the eighth day (D8) after ovulation, we used Pearson's correlation. Differences were considered significant at P < 0.05.

## Results

The three protocols used did not have an effect on the pre-ovulatory follicle diameter, ovulation rate, or the interval between inducer application and ovulation (P > 0.05), but did have an effect on the ovulation rate more than 48 h after induction (P = 0.03) (Table 1). Donkeys submitted to the protocol using 450 IU hCG applied at the *Hou Hai* acupoint (T3) had a higher ovulation rate (72.73%) more than 48 h after induction, which means, only 27.27% of ovulations occurred in the first 48 h after induction.

**Table 1:** Pre-ovulatory follicle, ovulation rate and moment of ovulation of the donkey with ovulation induced by subdose of the human chorionic gonadotropin in *Hou Hai* acupoint

**Variables**	**T1 (n = 11)** **1**	**T2 (n = 11)2**	**T3 (n = 11)3**	**P value**
Diameter pre-ovulatory follicle (mm)^4^	34.6 ± 4.6	35.9 ± 3.0	33.2 ± 41	0.318
Ovulation rate (%)^5^	100.00	90.91	100.00	0.368
Interval inducter and ovulation (h) ^4^	50.0 ± 39.9	46.8 ± 21.5	77.4 ± 28.0	0.058
Ovulation above 48h after induction (%)^5^	18.18^a^	36.36^ab^	72.73^b^	0.034

1 T1 = 1500 IU of hCG IV. ^2^ T2 = 450 IU of hCG IV. ^3^ T3 = 450 IU of hCG of *Hou Hai* acupoint. ^4^ Parametric data, refer to the mean ± standard deviation and were not influenced by the treatments, ANOVA (P >0.05). ^5^ Non-parametric data. ^ab^ There was difference by the Kruskal Wallis test (P <0.05).

The use of a subdose of hCG administered at either the false acupoint or the *Hou Hai* acupoint did not affect (P > 0.05) the mean diameter of the CL on days D0, D2, and D8. Similarly, the serum concentration of P_4_ eight days after ovulation was not affected by the induction protocols used (P > 0.05) (Table 2), but the protocols did affect the CL diameter on D4 (P = 0.04). Donkeys administered 450 IU of hCG at the *Hou Hai* acupoint (T3) had a larger CL diameter, which was similar to that of donkeys administered 450 IU (IV) in the false acupoint (T2), but higher than that of donkeys receiving the full dose of hCG by IV (Table 2). This suggests that commonly used doses for ovulation induction in donkeys are possibly too high.

**Table 2:** Development of the corpus luteum and serum progesterone concentration of donkey with ovulation induced by subdoses of human chorionic gonadotrophin in *Hou Hai* acupoint

**Variables**	**T1 (n = 11)** **1**	**T2 (n = 11)2**	**T3 (n = 11)3**	***P* value**
Corpus luteum on ovulation day (cm)	22.4 ± 7.3	23.3 ± 4.9	23.4 ± 4.8	0.91
Corpus luteum 2d after ovulation (cm)	25.9 ± 5.2	27.6 ± 3.7	29.6 ± 5.4	0.21
Corpus luteum 4d after ovulation (cm)	25.8 ± 4.4^a^	27.0 ± 4.5^ab^	30.7 ± 5.1^b^	0.04
Corpus luteum 8d after ovulation (cm)	27.3 ± 2.4	28.8 ± 4.7	28.5 ± 3.1	0.54
Progesterone concentration 8d (ng/mL)	7.65 ± 7.11	10.22 ± 9.45	13.63 ± 11.12	0.32

1 T1 = 1500 IU of hCG IV. ^2^ T2 = 450 IU of hCG IV. ^3^ T3 = 450 IU of hCG of *Hou Hai* acupoint. The data refer to the mean ± standard deviation. ^ab^ Different letters overwritten in the same line differ (P = 0.046) for the Tukey test

**Figure 1:**
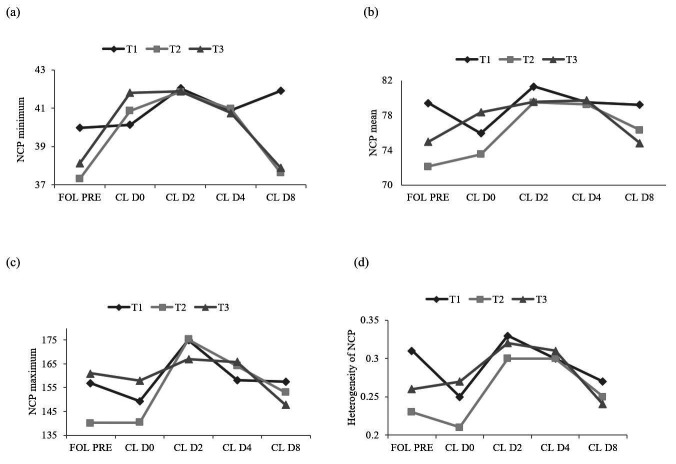
Minimum (a), medium (b), maximum (c) and heterogeneity (d) of the number of colored pixel (NCP) of the pre-ovulatory follicle (FOL PRE) and corpus luteum (CL) on days 0 (D0), 2 (D2), 4 (D4) and 8 (D8) after ovulation. T1 = 1,500 IU of hCG IV; T2 = 450 IU of hCG IV; T3 = 450 IU of hCG in *Hou Hai* acupoint. There was a statistical difference for CL D4 by the Tukey test (P < 0.05) (a). There was no difference between treatments for FOL PRE; CL D0; CL D2 and CL D4, ANOVA (P > 0.05) (b, c, d).

The administration of 1,500 IU of hCG IV (T1) resulted in higher blood perfusion of the CL on D8, as assessed by the minimum NCP (P < 0.05) (Figure 1a). However, the mean, maximum, and heterogeneity of number of colored pixel were similar between treatments for both the pre-ovulatory follicle and CL on D0, D2, D4, and D8 (P > 0.05), indicating that there was no significant effect of the hCG dose used, nor of the route of application (Figure 1b, c, d).

For donkeys receiving subdoses of hCG (450 IU) both at the false acupoint and at the *Hou Hai* acupoint, a positive correlation was found between mean NCP and P_4_ concentration on D8 (P < 0.05). However, there was no correlation between the same variables for the donkeys that received the full dose of hCG (1,500 IUI) by IV (T1), nor between the size of the CL and the concentration of P_4_. That is, the behavior of one of these variables did not influence the behavior of the other (P > 0.05).

## Discussion

The treatments resulted in similar patterns in the pre-ovulatory follicle diameter, ovulation rate, and the interval between induction and ovulation, indicating that the dose of 450 IU administered intravenously at the false acupoint, or at the *Hou Hai* acupoint can be used to induce ovulation in donkeys (Table 1). When using 450 IU of hCG, the mean diameter of the pre-ovulatory follicle was 34.5 mm, which is within the predicted range for the species, which may vary between 34 and 45 mm, for either induced or natural ovulation. Derar and Hussein (2011) obtained a mean diameter of 36 mm when evaluating follicular dynamics of donkeys during the estrous cycle, with spontaneous ovulation. Carluccio et al. (2007) previously obtained a mean diameter of the pre-ovulatory follicle of 38 mm by inducing ovulation of follicles with a diameter between 30 and 35 mm with 2,500 IU of hCG, which is a higher dose than that used in the present study.

Quaresma and Payan-Carreira (2015) reported a mean follicle diameter of 40.2 mm in single ovulations, 36.7 mm in double ovulations, and 38.6 mm in triple ovulations, when characterizing the estrous cycle of donkeys. This was similar to the results of Carluccio et al. (2017), who obtained pre-ovulatory follicle diameter of up to 45.1 mm in postpartum estrous cycles for this species, without using an ovulation inducer. Both studies found higher mean follicle diameter values than the one obtained in the present study. However, it is worth mentioning that in mares, the application of hCG anticipates the ovulation of pre-ovulatory follicles, which usually have a smaller diameter than follicles resulting from spontaneous ovulations, as hCG accelerates follicular maturation and ovulation, as described by Cuervo-Arango and Newcombe (2008). The same is likely the case in donkeys, since hCG acts in the same way, independent of the species.

The use of hCG as an ovulation inducer promotes high ovulation rates, as demonstrated in the present study (Table 1), and in previous studies using higher doses of hCG in the same species (Carluccio et al., 2007) or in mares (Morel et al., 2010). The objective of induction is the prediction of the time of ovulation, and therefore differences between the experimental groups regarding the time interval between induction and ovulation were also considered in this study. We found no significant effect of our treatments, although hCG appeared to meet this criterion better. According to Samper (2008), the mean response time to hCG is around 36 hours, ranging from 12 to 48 h when using doses between 1,500 to 3,300 IU IM or IV.

In fact, in the present study, most ovulations (81.82%) in donkeys that received 1,500 IU of hCG IV (T1) occurred before 48 h (P < 0.05). However, in the donkeys that received 450 IU at the *Hou Hai* acupoint (T3), despite having a 100% ovulation rate, only 27.27% of ovulations occurred during the first 48 h after induction (Table 1). This result differs from that found by Carluccio et al. (2007), who found that 91.7% of predicted ovulations occurred within the first 48 hours after induction, although these authors administered a higher hCG dose (2,500 IU).

The protocols used in the present study were effective for inducing ovulation, but should be used when using natural mating or artificial insemination with fresh semen, due to the greater sperm viability in the reproductive tract of donkeys. The alternative for using cooled semen would be to perform hCG treatment 12-24 h after detection of follicles that are ≥ 30 mm in size.

The use of a subdose of hCG at the false acupoint (IV) or *Hou Hai* acupoint as an ovulation inducer was as effective in promoting the development of the CL as the dose of 1,500 IU applied by the IV route, as demonstrated by the diameter of the CL obtained from the day of ovulation (D0) to D8, as well as by serum P_4_ concentrations (Table 2). In addition, donkeys treated with 450 IU of hCG at the *Hou Hai* acupoint had the largest diameter of CL on D4 (P = 0.04), which may have been caused by the longer interval between induction and ovulation, which may have resulted in larger follicles and consequently the corresponding formation of larger CL.

The maximum CL diameter found in donkeys was 30.7 ± 5.1 mm on D4, which differs from the results reported by Conceição et al. (2009), who found a maximum diameter of 26.2 ± 4.4 mm on D5 for simple ovulations following the luteal development of donkeys. Derar and Hussein (2011), when evaluating the estrous cycle of donkeys, reported a maximum CL diameter of 26.77 ± 1.28 mm on D13, with the CL regressing until D17. These results demonstrate the efficiency of the use of subdoses of hCG in the ovulation protocol applied at the *Hou Hai* acupoint in donkeys, as well as for promoting the adequate development of CL when compared to CL formed from spontaneous ovulations, and those formed from induced ovulations with a higher dose of IV hCG (1,500 IU).

The concentration of P_4_ on the eighth day after ovulation was similar between treatments, and similar to results obtained by Carluccio et al. (2008), who evaluated 22 donkeys, and reported a mean serum P_4_ concentration of 9.1 ng.mL^-1^. This indicates the possible functionality of CL, since, according to Grizendi and Fernandes (2015) and Sieme et al. (2016), P_4_ concentrations below 2 ng.mL^-1^ are characteristic of luteal insufficiency in mares and donkeys. In this study, we obtained serum P_4_ concentrations below 2 ng.mL^-1^ in four donkeys, and serum P_4_ concentrations below 1 ng.mL^-1^ in only one donkey. This is not uncommon, and, according to Conceição et al. (2009), P_4_ concentrations below 1 ng.mL^-1^ are characteristic of the estrogenic phase. These results suggest that, considering only progesterone production, 15.15% of the donkeys evaluated had non-functional CL.

However, evaluation of CL diameter and P_4_ concentration alone does not allow the determination of CL functionality (Carluccio et al., 2008), although some studies indicate that the larger the diameter, the higher the hormone production (in this case, P_4_). To determine CL functionality, it is necessary to associate the echogenicity and blood perfusion characteristics of CL, and the serum P_4_ concentration, since the vascularization of the CL and the serum P_4_ concentration increase in the days after ovulation, thus enabling the prediction of the CL functionality.

Thus, in this study, we evaluated the vascularization of the ovarian structures, the pre-ovulatory follicle, and the CL, using the average, maximum, and minimum number of colored pixel (NCP), as well as the NCP heterogeneity, obtained by Doppler ultrasound examinations. According to Díaz-Duran et al. (2017), after ovulation, neovascularization is essential to draw substrates to the luteal cells, which are responsible for the production and subsequent secretion of P_4_. As such, it is important to use methods that make the evaluation of CL vascularization more objective, as such methods tend to improve the results of the reproductive biotechniques used.

There was a significant effect of treatments on CL tissue changes between D4 and D8 regarding the minimum number of pixels (P < 0.05). The donkeys receiving 1,500 IU of IV hCG (T1) had a higher minimum NCP (Figure 1a). However, the same patterns were observed regarding theaf mean and maximum NCPs, and for the NCP heterogeneity in the three treatments (P > 0.05) (Figure 1b, c, d), with values reduced from the pre-ovulatory follicle and CL on D0. Maximum values were obtained on D2, which decreased until D8, indicating that the therapy used in the present study did not improve luteal blood flow, regardless of dose or route of application.

The results of the present study corroborate those of Romano et al. (2015), who evaluated the hemodynamics of mares treated with hCG and did not find a significant effect of hCG treatment. These authors concluded that the use of hCG as an ovulation inducer does not affect the serum concentration of P_4_ in either donkeys or mares. However, Gastal et al. (2006) reported a progressive increase in follicular vascularization at the time of ovulation induction after hCG treatment, with a sudden reduction in the four hours before ovulation, detected at one-hour intervals. The divergence between the results obtained in these studies suggests that other studies on the hemodynamics of the pre-ovulatory follicle and CL of donkeys, treated with an ovulation inducer or not, should be performed. Such studies will contribute to our understanding of the reproductive physiology of the species.

The results of this study demonstrate that, in addition to the possibility of evaluating the CL activity through its diameter and P_4_ production, it is possible to study the development of the follicle echographically from the development of the follicle to the formation of CL in the ovarian tissue, and to correlate these with the endometrial modifications. This allows us identify what would be a normal CL, associating its morphology and function. In this study, when evaluating the association between CL diameter, P_4_ concentration, and NCP, a positive correlation was found only between the mean NCP and P_4_ concentration on D8 in donkeys receiving hCG, regardless of the application route. Studies using mares also found a positive correlation between blood flow and P_4_ concentration (Ginther et al., 2007) although studies using Doppler ultrasonography for evaluation in donkey species are rare.

Although some studies found a positive correlation between CL size and serum P_4_ concentration (Arruda et al., 2001), in the present study, there was no correlation between these variables. That is, the increase in CL area did not have an effect on P_4_ concentration in the bloodstream. This was also found by Rodrigues et al., (2014), who reported that, in their study, the correlation between these variables was non-existent. As Nagy et al. (2004) point out, other individual factors may affect P_4_ concentrations, such as weight, age, metabolism and amount of hormone receptors.

## Conclusion

The association of CL diameter with P_4_ concentration and tissue changes in NCP allows us to infer that the CL formed were functional and capable of maintaining gestation, regardless of the dose of hCG used and the route of application. This is a noteworthy result, since the use of subdoses of hCG to induce ovulation makes it possible to reduce the cost of the protocol, and reduces the risks of anti-hCG antibody formation, in addition to promoting ovulation effectively. The use of Doppler ultrasonography is pertinent, because this makes it possible to predict the functionality of CL in real time based on their blood flow, which may be associated with the diameter.
